# From Our Correspondents

**Published:** 1984-01

**Authors:** 


### From Our Correspondents

**Deputising Services**

General practitioners contract to provide general medical services for their patients twenty-four hours a day and every day of the year. Few are now able to achieve this degree of service to their patients and many different means of allowing adequate time off duty have been devised. The traditional way has been to link with nearby colleagues and arrange a rota with them. With the increase in group partnerships and health centres over the last 20 years the number of doctors taking part in such rotas has steadily increased, so that sometimes one doctor may be covering ten doctors' patients and be on call for about 20,000 patients at night time or at week-ends.

In large cities, such as Bristol, it has become commercially viable to organise a doctors' deputising service. Recently such deputising services have come under considerable criticism which centres around their inability to provide a service that is supposedly available from small rotas of doctors. Criticisms come from two main sources: the public and our hospital colleagues.

Patients' criticisms are few and usually relate to the delay in obtaining assistance or unhappiness with what the doctor prescribed. Hospital doctors' criticisms centre around 'needless' admissions, or admissions without adequate background information.

Over half of the city's 220 general practitioners use the service to some extent and they all seem to be happy with the service that is provided. What then are the problems?

The deputising service encourages irresponsible use of doctors' time. Practices that use the service totally for their out-of-hours calls have a higher than average call-out rate. The service does not attempt to advise, when patients call for help; a doctor is always sent.

The second problem relates to the service that is provided. In some schemes the quality of the locums is poor and doctors receive little or no encouragement to keep patients at home and arrange for a revisit; it is much more convenient to arrange an admission.

Quality of care in general practice is extremely difficult to measure. Is it good to prescribe readily or better to encourage patients to manage simple illnesses themselves? Is it good to encourage patients to stop smoking or better to keep them happy by not mentioning it; better to prescribe slimming aids or to refuse to do so. The use of deputising services by a general practitioner falls into a similar category. Some will find that using such a service is the only way they feel they can survive (for instance, single handed practices in the city); others will prefer to spend their money on such a service to ensure they can devote their evenings and week-ends to non-medical pursuits.

Many, though, will maintain that using a rota of a small group of well-known local colleagues provides a much better service to their patients whilst enabling the doctor to have adequate off duty.

M.J.W.

**Paper**

It may be a sign of advancing age, but I get increasingly annoyed at the vast amount of paper that is involved in hospital treatment. Every ancillary department seems to delight in producing its own request form, and then has special forms for progress and assessment. In the case of speech therapy these seem to be increasingly incomprehensible! An in-patient collects fluid balance charts, neurological observations, records of blood pressure, T.P.R., urine and blood tests for sugar, separate sheets for sticking on reports from the different branches of pathology, and X-ray reports. If a patient is having a baby the notes swell like the mother's abdomen.

Out-patient letters from some of my colleagues often cover an A4 sheet, and some recent surgical 'summaries' I have received cover two such sheets. Communications between departments seem now of necessity to be typed on a large sheet of paper with copies to all concerned. I delight in consigning most of them to the waste paper basket, or returning them with a comment on the bottom 'I agree' or some such remark. Many such long letters are a poor substitute for talking to the individual concerned, and often emanate from colleagues who never come to lunch or to meetings which are the best venue for deciding on shared problems.

H.G.M.

**Clinical Budgets**

The previous remarks are partly relevant to this subject. Southmead has been chosen for an attempt to cost treatments of common surgical and medical conditions. Humphrey White and I are working with a group of accountants trying to estimate the average stay, theatre usage, consumption for drugs, use of ancillary departments and disposable materials of conditions such as hernia repair and coronary thrombosis. We have pointed out the tremendous differences that can occur in the costs of treatment according to age, severity, social background and so forth. There are so many variables in the patients we have to admit, and for instance one diabetic may be treated in a few days with insulin and intravenous fluid, whereas the next one may need complicated antibiotic treatment for kidney infection, several X-rays and perhaps surgical intervention. By contrast it is relatively easy to work out the cost of a blood test, an X-ray, a standard treatment in physiotherapy, a catheterisation or a drug. However, I think to attempt clinical budgeting is worthwhile, if only to make us all look critically at our use of resources, and hence to think of economies. The study is attracting considerable interest in the profession, and already I have been interviewed on the P.M. programme of the B.B.C.!

H.G.M.

**Scanners**

In the early 1970's, a new system of X-ray examination was invented by a team of research scientists working for E.M.I. Ltd., called computerised tomography (C.T.). The team was led by Godfrey Hounsfield, later to receive a Nobel prize and a knighthood for his services to science and medicine. The system was applied at first to the examination of brain only, as the part being X-rayed had to be surrounded by a water bag and each 'slice' took four and a half minutes to examine, which excluded any area subject to movement. Frenchay Hospital were lucky enough to receive one of the first machines in 1974, and it was apparent from early on that the new technique was going to revolutionise the approach to disease of the central nervous system, which of course it was quick to do.

Since those early days, the technique has been developed and improved, so that the new machines produce pictures of superb quality and detail that are astonishing when compared with the earlier results. The technique is now applicable to the whole body, as is well known, and considerable information of clinical and diagnostic value is available, not only in the head, but now also in the chest, abdomen, spine and elsewhere, so that there are many specialties able to receive helpful information in areas previously blind to diagnostic techniques. 'Slices' can now be just a few mms thick, exposures just a few seconds long, so that moving parts can be stilled, and superb detail obtained. Such is the speed of new technology that progress in a decade is truly staggering, which may have to be seen to be believed.

The early E.M.I. machines sold like hot cakes World Wide, but like many pace-setters before them, financial problems soon beset them, and international competition on a grand scale set in. In the early days Japan assembled C.T. machines under licence from E.M.I., but as in other manufacturing industries the scene was soon to change. The C.T. part of E.M.I., when finances eventually failed, was bought by I.G.E., an American company, and the Japanese also then started to produce their own machines. Nowadays we see Toshiba and Hitachi nameplates on C.T. scanners in use in this country and elsewhere in Europe. In Japan, the present home of high technology, there are now 2500 C.T. scanners and it is an interesting comment overheard recently in Tokyo that no Japanese would choose to go to a hospital unless it was equipped with a C.T. machine! I take it that the man in the street in Japan regards the C.T. scanner as part of the diagnostic armamentarium he would expect to find in any well equipped hospital. By comparison it makes an interesting and perhaps challenging comparison that in the U.K. there are about 80 scanners installed, and quite a high proportion of these have been bought by public subscription or charitable bodies.

In the South West of England for example, there are at present six scanners, only two of which were bought out of Regional funds. The others were all provided out of public subscription, W.P.A. or D.H.S.S. funds. And furthermore the S.W. Region is one of the better provided regions in the country as far as the provision of these facilities goes. It is heartening therefore to learn that three new whole body scanners will be provided during the year 1984. One will be installed at Frenchay Hospital to replace their ageing head machine, which has examined over 50,000 heads in its 10 years of life. A further one will be installed at the Royal Infirmary as a new development in the Diagnostic X-ray Department and a third one will be installed as a new development at Truro, where public subscription recently topped £300,000 as a contribution towards the slightly greater amount out of Regional funds. It was with the aid of a special grant from the Region that these new scanners will materialise. They will be of immense value to the hospitals and the patients that they serve. Other machines in the Region will soon need to be replaced and new ones installed elsewhere. There is a strong case for the attitude that every major district hospital needs a C.T. scanner. Personnel also need to be trained to use them, and the extra cost should be accepted as part of the new technological age we live in. The pay-off is better diagnostic results for the patient, better treatment that will follow, and shorter in-patient stay in hospital. We may have to follow the Japanese in understanding the value of C.T. and using it to proper advantage.

J.L.G.T.

**Anastomosing Drains**

For the past two months one corner of Southmead Hospital could have been mistaken for the Flanders battlefield. Trenches of all sizes and shapes have criss-crossed the roads around the pathology laboratory and to the observant have provided endless interest. It might be thought that replacing the main drains would be a simple procedure similar to a major arterial graft. Not so; the segment being replaced mimicked at least the coeliac axis and its major tributaries. All the main bifurcations required new manholes with ingenious concrete junction boxes, created on the spot around huge plastic moulds. All the digging was performed by one large tractor with a preying-mantis scoop at one end and a fork-lift hydraulic shovel at the other. The expertise of the operator never failed to fascinate, whether he was gently returning the last heap of rubble into a refilled drain or using his scoop as a crane to lower a vast concrete pipe into the 'aorta'.

To the Urologist or Vascular Surgeon the anastomoses of pipes and their roofdrain collaterals was a logical and planned operation and when one day a coronary 'venous' bypass, in the shape of a hose and pump arrived, this seemed the obvious solution to a tricky occlusion. The logistics of all this effort were also fascinating. A never ceasing flow of dumper trucks, small and large lorries either taking soil away or bringing concrete, new ballast or Macadam, always seemed to be in the right place at the right time. The sheer efficiency of the whole operation was impressive and despite an ever increasing patchwork of dug and re-dug trenches the traffic flow through a vital major internal road was only mildly disturbed. The only major complication to this efficient operation was rupture of the gas main which added a further series of trenches, mysterious gas lead detectors and a warning sign, 'No Naked Lights' worrying to the inveterate pipe smoker discarding his matches.

Apart from providing endless entertainment, this drain replacement programme with only phase 1 completed, is a reminder that so much of the cost of running a hospital is unseen and not newsworthy. It is easy to raise money for emotional causes such as leukaemic research, kidney machines and monitoring equipment for neonates but no one attempts to raise money for the basic services.

How about starting a 'New Drains for Southmead Appeal'—sign your own pipe?

C.R.T.

**Long Live the NHS**

On December 5th 1983 *The Times* gave prominence to an article entitled, 'Time to Sell off the NHS'. It must be among the worst articles of its genre ever written. Out came all the hoary old platitudes and half-truths about the mismanagement of and wastefulness in the Health Services. 'If only doctors would do some work instead of spending all their time in private practice', pontificated the author. The article was so obviously inconsistent in its theme and argument that it is amazing that it got past the editor's blue pencil.

But who was the fount of all wisdom, the saviour of the NHS? None other than David Hart (novelist and political adviser). 'Who?' I hear you ask? According to the *Daily Mirror* (8th December) he is a discharged bankrupt who advises Mrs. Thatcher on economic and social affairs. I have only their word for it, but of such are experts made!

Such misguided multiwarheaded literary missiles are, of course, impossible to rebut concisely. Perhaps that is why they are printed in the first place. In a letter to the editor of *The Times* on 7th December, your correspondent tried to redress the balance by pointing out one aspect of the credit of the NHS. Since 'Good news is not news' it was a forlorn hope to think that it would be published. Sure enough, on December 13th, 'the editor regrets' note arrived. Up to the time of writing no corrective has appeared, so for what it's worth, here is mine. Long live the NHS!

Miss A. was 19 when she became pregnant. Unemployed, overweight and a heavy smoker, she could never remember to take 'the Pill' regularly. By the time she first attended the hospital ante-natal clinic at about 22 weeks of pregnancy, things were already beginning to go wrong. Her blood pressure was much higher than it should have been; she was putting on too much weight, and the baby seemed smaller than a best guess suggested it should have been.

She refused admission to get things sorted out, and her unemployed boyfriend was abusive to the staff who suggested it. Things went from bad to worse, and by 26 weeks of pregnancy her blood pressure had reached crisis level. With great reluctance she agreed with her G.P. that she should be admitted. Her sullenness was only matched by her boyfriend's continuing abusiveness. Their confidence was, however, gradually won and it was obvious that their earlier behaviour was due to their terror of hospitals. Her state of health continued to deterioriate directly as a result of her pregnancy, and she required close observation day and night. It was thought extremely unlikely that the baby could survive.

About 10 days after her admission she suddenly began to show evidence that bleeding was occurring behind and within the placenta. Left for much longer, the baby was likely to die. It was 10p.m. on a Sunday evening. The senior consultant obstetrician in charge of her care was called, bringing with him the senior physician with whom he had been sharing care. The questions were: could the baby be saved; was it too small and too sick to survive?

After discussions with the mother and her partner, the baby was delivered by caesarean section (carried out under general anaesthesia provided by a consultant anaesthetist) into the hands of a waiting consultant paediatrician. By 1 a.m. it was becoming clearer that the baby had a fair chance of survival. The mother's condition had already improved. Both mother and baby did well.

Mrs. B, the wife of a taxi-driver, had lost four babies before birth as a result of recurrent catastrophic haemorrhages at various stages of pregnancy. No specific cause could be found. Desperately anxious to have a child, she became pregnant again. She was observed intensively throughout the pregnancy, much of it in hospital, and with treatment all seemed to be progressing satisfactorily.

It was decided that she should be delivered at 37 weeks of pregnancy. At 1 a.m. on the Sunday, three days before her scheduled data of delivery, she was wakened by abdominal pain and called the staff-nurse on night duty. She was bleeding once more and already the baby was showing signs of distress! A crash call was made to theatre and to the consultant in charge. He was in the hospital within ten minutes, by which time the baby had been born for three. Only seven minutes passed from the patient calling the nurse to delivery by Caesarean section. Mother and baby did well.

Where did all this take place? Within the NHS of course. But it is not a health service one would recognise from the unbalanced intemperate polemic of David Hart (novelist and political adviser—*The Times*, 5th December, 1983). It is, however, a health service in which we can still have pride, despite all its undoubted problems. Those women received such care because they needed it, not because they were paying for it item by item. Long live the NHS!

G.M.S.

### Ham Green Hospital

**Post Graduate Education**

Meetings for both General Practitioners and Hospital Doctors are now being held every Friday lunch time (at 1.10 p.m.) in Lester House (Doctors Residence) with a Clinical Meeting (with Case Presentations) on the 3rd Friday. The Area Chest Meeting is held on the 4th Friday at 3 p.m. in the Committee Room.

**Symposium—Relief of Pain**

A Study Day was held on 18th November attended by about 60 doctors and nurses to discuss the various aspects of Pain Relief. The session was opened by Dr. G. Mitcheson, a General Practitioner, who discussed the drugs he uses on first seeing a patient. He was followed by Dr. E. Walsh, Director of the Pain Clinic at Southmead Hospital who discussed specialized treatment. After lunch we had an interesting talk by Mr. Ken Johnson, Senior Social Worker who talked on the social, economic and financial aspects of pain and drew on many of his case histories to illustrate how important these aspects were in the overall relief of pain. The Final Session was taken by Dr Ian Capstick, Director of St Peter's Hospital, who told us that regular dosage of drugs was essential, only occasionally were injections necessary and even then these could be given through an indwelling cannula. We should not be afraid to give adequate drug therapy to achieve pain relief. We ended with a Panel Discussion and I'm sure came away feeling not only, 'could we do better, but we would do better in relieving pain in the future and that close liaison between the patient, the family, the nurse, and the doctor, and others would help to achieve this aim.

D.W.W.

**The De Snoo Prize**

On 1st October 1983 Dr. Peter M. Dunn, Reader in Child Health and Perinatal Medicine at the University of Bristol, received the De Snoo medal and prize from the hands of Mrs. De Snoo at a meeting of the Dutch Obstetric and Gynaecological Society in Utrecht, Holland. This prize of the De Snoo Foundation is awarded every three years alternately to a clinician or to a basic scientist who has made outstanding contributions in the field of human reproduction. The Foundation was created in 1949 in memory of Professor Klaus De Snoo who was the Doyen of Dutch Obstetrics and Gynaecology during much of the first half of the present century and was Professor in this discipline in the University of Utrecht from 1927–47. This, the 16th award, marks the first occasion that the prize has been awarded either to a paediatrician or to a doctor from the United Kingdom.

D McCOY

**Peripatetic Correspondent**

Roger Harman recently travelled by taxi to act as external examiner at the Institute of Dermatology. He was almost late because the taxi was held up behind a massive pantechnicon which seemed to be progressing erratically because of some mechanical defect. To pass the time, he chatted to the taxi driver about the purpose of his trip, and about the fact that the vehicle ahead seemed to have a slipping clutch. Shortly afterwards the monstrous machine ground to a complete standstill, bringing traffic to a halt. The driver leapt down from his cab and came to apologise 'Sorry mate, the clutch has gone'. The taxi driver turned to the dermatological examiner with a wicked wink—'I don't think those students will have a chance against you today—cor, fancy diagnosing clutch trouble without even taking the covers off'.

J. L. BURTON

